# The Therapeutic Potential of Natural Dietary Flavonoids against SARS-CoV-2 Infection

**DOI:** 10.3390/nu15153443

**Published:** 2023-08-03

**Authors:** Zhonglei Wang, Liyan Yang

**Affiliations:** 1 Key Laboratory of Green Natural Products and Pharmaceutical Intermediates in Colleges and Universities of Shandong Province, School of Chemistry and Chemical Engineering, Qufu Normal University, Qufu 273165, China; 2 School of Pharmaceutical Sciences, Key Laboratory of Bioorganic Phosphorus, Chemistry & Chemical Biology (Ministry of Education), Tsinghua University, Beijing 100084, China; 3 School of Physics and Physical Engineering, Qufu Normal University, Qufu 273165, China; 4 Beijing National Laboratory for Molecular Sciences, Institute of Chemistry, Chinese Academy of Sciences, Beijing 100190, China

**Keywords:** COVID-19, SARS-CoV-2, natural dietary flavonoids, broad-spectrum antiviral activities, multi-organ protective capacity, combination therapy, lead optimization

## Abstract

The exploration of non-toxic and cost-effective dietary components, such as epigallocatechin 3-gallate and myricetin, for health improvement and disease treatment has recently attracted substantial research attention. The recent COVID-19 pandemic has provided a unique opportunity for the investigation and identification of dietary components capable of treating viral infections, as well as gathering the evidence needed to address the major challenges presented by public health emergencies. Dietary components hold great potential as a starting point for further drug development for the treatment and prevention of SARS-CoV-2 infection owing to their good safety, broad-spectrum antiviral activities, and multi-organ protective capacity. Here, we review current knowledge of the characteristics—chemical composition, bioactive properties, and putative mechanisms of action—of natural bioactive dietary flavonoids with the potential for targeting SARS-CoV-2 and its variants. Notably, we present promising strategies (combination therapy, lead optimization, and drug delivery) to overcome the inherent deficiencies of natural dietary flavonoids, such as limited bioavailability and poor stability.

## 1. Introduction

The outbreak of coronavirus disease 2019 (COVID-19) caused by severe acute respiratory syndrome coronavirus 2 (SARS-CoV-2), the deadliest virus since the 1918 influenza virus, has posed a serious threat to global health security [1]. Tremendous research efforts have been undertaken, aiming at controlling and/or treating SARS-CoV-2 infection [2,3,4]. To date, several small-molecule antivirals (remdesivir, ritonavir-boosted nirmatrelvir, and molnupiravir), vaccines, and monoclonal antibodies have been approved or authorized by the Food and Drug Administration (FDA) of the United States of America for the treatment of COVID-19 [5,6,7,8]. Although the pandemic appears to be on a downward trend, the potential emergence of new SARS-CoV-2 variants still represents a threat to humans, given their intrinsic transmissibility, immune escape, virulence, and susceptibility to available treatments [9,10,11]. Taking virulence as an example, if it is assumed that the mortality rate among SARS-CoV-2-infected individuals is similar to that for seasonal influenza, we can expect the annual burden of future influenza to be twice that of previous influenza. The additional burden associated with “long COVID” (e.g., respiratory symptoms and cognitive dysfunction) may also be non-negligible [12,13]. Accordingly, an inexpensive, convenient, and rapidly up-scalable response model is required to address future coronavirus pandemics.

**Figure 1:**
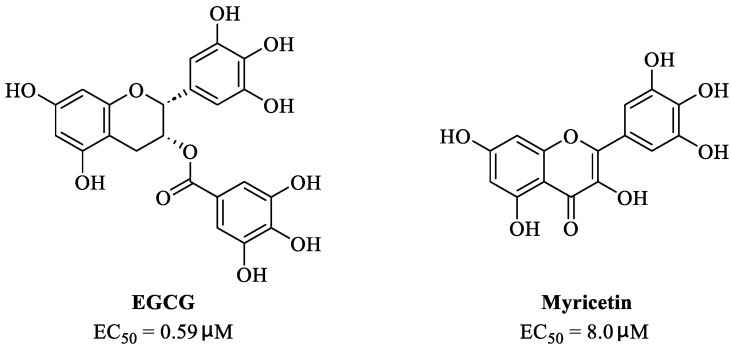
The chemical structures of epigallocatechin 3-gallate (EGCG) and myricetin.

Natural products (including herbal medicine) play an irreplaceable role in the treatment of SARS-CoV-2 infection [14,15]. Increasing evidence supports that many functional foods and nutraceuticals have potential for use in the prevention and treatment of viral infections [16]. In recent years, flavonoids have attracted much attention from pharmaceutical chemists and organic chemists due to their efficiency and low toxicity for health improvement and disease treatment. Their active components, such as epigallocatechin 3-gallate (EGCG) and myricetin (Figure 1), have drawn considerable attention as potential agents for COVID-19 treatment owing to their multitargeting potential (SARS-CoV-2 M^pro^, angiotensin-converting enzyme 2 [ACE2, the primary target of SARS-CoV-2 in host cells], and RNA-dependent RNA polymerase [RdRp, an essential enzyme in RNA viruses, which is a key player driving the viral replication and transcription machinery], among other targets), broad-spectrum activities, and low toxicity [17,18]. In this review, we summarize the characteristics of natural dietary flavonoids, including their bioactive properties and potential mechanisms of action, associated with the prevention and treatment of COVID-19, and discuss strategies aiming at improving their bioavailability, chemical stability, and delivery. Finally, we present promising strategies (combination therapy and lead optimization) for overcoming the inherent shortcomings (limited bioavailability and poor chemical stability) of natural dietary flavonoids as therapeutics for SARS-CoV-2 infection.

## 2. Epigallocatechin 3-Gallate—A Green Tea-Derived, Multitargeting, Anti-SARS-CoV-2 Therapeutic Candidate

**Figure 2:**
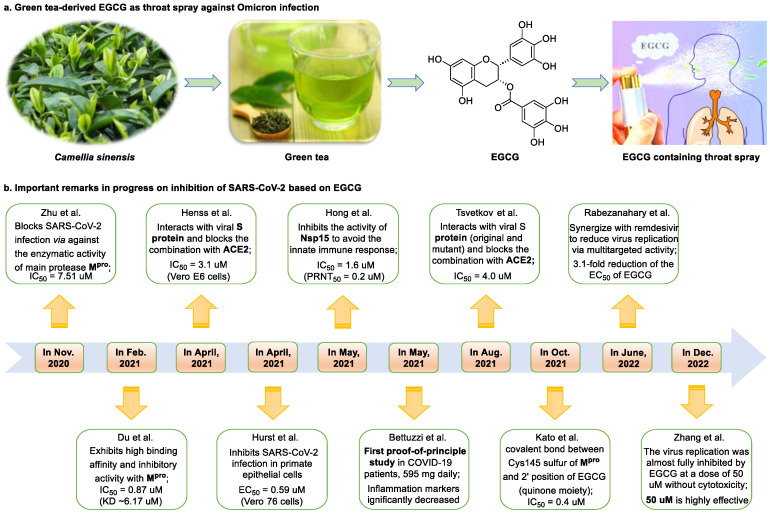
Epigallocatechin 3-gallate is a green tea-derived, multitargeting, anti-SARS-CoV-2 therapeutic candidate. (**a**) Epigallocatechin 3-gallate (EGCG), isolated from *Camellia sinensis*, has potential for development as a therapeutic throat spray for Omicron infection. (**b**) Important discoveries relating to the multi-target effects of EGCG against SARS-CoV-2. Data from references [17,34,35,37,38,39,40,41,42,43].

Epigallocatechin 3-gallate (EGCG), a nutritional supplement with promising health-beneficial effects isolated from green tea (*Camellia sinensis*) (Figure 2a), has long been investigated for its potential as supplementation therapy for the prevention of numerous disorders, including cancer [19] and cardiovascular [20], metabolic [21], neurodegenerative [22], and infectious diseases [23]. For instance, Polyphenon E^®^, comprising >65% EGCG, is a standardized preparation of green tea catechins approved by the US FDA in 2006 for the treatment of external genital and perianal warts [24]. Polyphenon E has an excellent safety and tolerability profile, an essential characteristic allowing for the extensive use of EGCG [24]. The green tea catechin palmitate (comprising 50% EGCG), an oil-soluble green tea extract, was approved by the US FDA in 2019 as a safe dietary ingredient [25]. In vitro, EGCG has highly promising broad-spectrum antiviral activity, including against Zika virus (half-maximal effective concentration [EC_50_] = 21.4 μM) [26], hepatitis B virus (half-maximal inhibitory concentration [IC_50_] = 0.11 μM) [27], Japanese encephalitis virus (IC_50_ = 7.0 μM) [28], human coronavirus (HCoV) 229E (IC_50_ = 0.77 μM) [29], human coronavirus OC43 (IC_50_ = 0.49 μM) [29], Middle East respiratory syndrome (MERS)-CoV (IC_50_ = 8.4 μM) [30], and SARS-CoV (IC_50_ = 1.5 μM) [30]. Given its excellent safety and broad-spectrum antiviral activities, EGCG may contribute to immediate clinical solutions for COVID-19 treatment.

Many studies have reported the impressive effects of EGCG against SARS-CoV-2 in vitro (Figure 2b). For example, Hurst et al. [17] demonstrated that EGCG blocks SARS-CoV-2 infection in Vero 76 cells (EC_50_ = 0.59 µM), while displaying only mild toxicity (selectivity index [SI] = 8.5). The 3C-like protease (3CL^pro^, also known as M^pro^) is highly conserved among coronaviruses, including SARS-CoV-2 [31,32]. Given its essential role in viral replication and transcription, M^pro^ represents a promising therapeutic target against coronavirus infection [33]. Du et al. [34] showed that EGCG is a potent inhibitor of M^pro^, with an IC_50_ of 0.87 μM. Surface plasmon resonance binding experiments demonstrated that EGCG has a high binding affinity for M^pro^, with a dissociation constant (KD) of 6.17 μM. Similarly, Zhu et al. [35] reported that EGCG inhibits M^pro^ activity, with an IC_50_ value of 7.51 µM. Furthermore, Ngwe Tun et al. [36] indicated that EGCG is highly effective at inhibiting SARS-CoV-2 replication (IC_50_ = 6.5 μM) in Vero E6 cells and with minimal toxicity (SI >154). Mechanistically, the authors further demonstrated that EGCG blocks SARS-CoV-2 replication at both the entry and post-entry stages of infection, and also inhibits SARS-CoV-2 M^pro^ activity.

**Figure 3:**
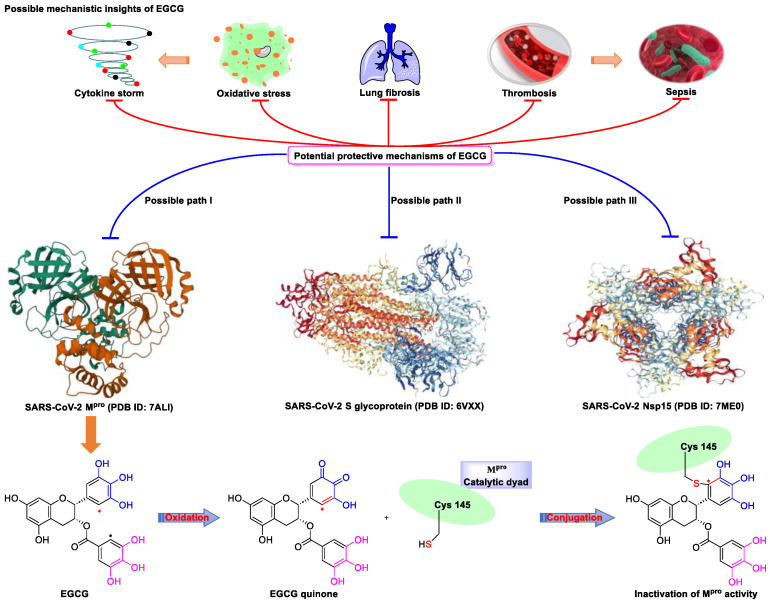
Proposed multi-target (S protein, Nsp15, and M^pro^) mechanism of action of EGCG against SARS-CoV-2: inhibition of oxidative stress, cytokine storm, lung fibrosis, thrombosis, and sepsis injury in SARS-CoV-2 infection. Oxidized EGCG is first recognized by the catalytic site of M^pro^, which is followed by the covalent bonding between the *α,β*-unsaturated carbonyl moiety of EGCG (serves as an electrophile) and Cys145 of M^pro^ (serves as a nucleophile).

Meanwhile, Kato et al. [37] showed that EGCG strongly inhibits the activity of M^pro^ (IC_50_ = 0.4 μM) via the formation of a covalent bond between Cys145 of the enzyme and the 2′-position of EGCG (Figure 3). Tsvetkov et al. [38] showed that partial EGCG treatment is highly effective at suppressing viral replication (IC_50_ = 4.0 μM, SI = 6) by interfering with the binding between ACE2 and SARS-CoV-2 spike (S) protein. Similarly, Henss et al. [39] reported that EGCG inhibits SARS-CoV-2 infection (IC_50_ = 3.1 µM, SI > 11.6) in Vero E6 cells through binding at the SARS-CoV-2 S–ACE2 interface. SARS-CoV-2 endoribonuclease NendoU (NSP15), a uridine-specific endoribonuclease used by the virus to avoid the innate immune response, is considered a compelling drug target [44]. Hong et al. [40] showed that EGCG strongly inhibits the activity of NSP15, with an IC_50_ value of 1.6 μM. In the same study, the authors investigated the neutralizing effect of EGCG against SARS-CoV-2 and obtained a promising result (half neutralization effect concentration [PRNT_50_] = 0.2 μM). The above findings regarding the efficacy of EGCG appear to be generalized, indicative of the therapeutic potential of EGCG for the treatment of COVID-19. Meanwhile, combination drug therapy may offer additional advantages [45]. Rabezanahary et al. [41] revealed that the combination of EGCG (15.6 µM) and remdesivir (1.25 µM), the first FDA-approved inhibitor of SARS-CoV-2 RdRp, exerts a significant synergistic effect (3.1-fold reduction in the EC_50_ of EGCG for RdRp) in Vero E6 cells through multitargeting activity.

Bettuzzi et al. [42] conducted a 15-day, proof-of-principle study to evaluate the anti-SARS-CoV-2 efficacy of EGCG and catechins (two sessions of inhalation plus three capsules daily; total EGCG: 595 mg; total catechins: 840 mg) in 10 non-hospitalized SARS-CoV-2 swab-positive patients. All patients were asymptomatic within 7 to 15 days of starting treatment, while the levels of inflammation markers significantly decreased. No observable adverse events with the EGCG treatment were reported. Additionally, compared with wild-type or Delta strains, Omicron strains have greater replicative capacity in the upper respiratory tract, increasing the likelihood of viral release during breathing; this characteristic may help explain the enhanced transmission of Omicron strains via airborne routes [46]. Yang et al. [47] demonstrated that after drinking two to three cups of green tea, the levels of EGCG in saliva ranged from 4.8 to 22 µg/mL (equivalent to 8.7–39.9 µM), which was two orders of magnitude higher than those in plasma. For cancer prevention, it is recommended that humans consume six cups of green tea daily [47]; accordingly, high doses of EGCG (up to 79.8 μM in saliva) are likely to be safe and may prove highly effective in controlling Omicron infection. Similarly, Furushima et al. [48] investigated the oral retention of catechins in healthy adults after the intake of a beverage (40 mL) containing 73.4 mg of catechins. They found that the average concentrations of EGCG in the oral cavity were approximately 156.3, 58.4, and 50.5 μM at 10, 40, and 60 min, respectively. These findings support the potential value of EGCG as a supplementation therapy for patients infected with an Omicron variant.

SARS-CoV-2 infection can have long-term effects on the lungs as well as on multiple extrapulmonary tissues and organs, while EGCG exerts unique multi-organ protective effects. For example, EGCG plays an important neuroprotective role following traumatic brain injury (through the activation of the adenosine monophosphate-activated protein kinase pathway), [49] ameliorates liver injury secondary to *Pseudomonas aeruginosa* pneumonia (via upregulating nuclear receptor activation), [50] protects cardiomyocytes against hypoxia–reperfusion injury (via potently inhibiting the self-cleavage of OMA1), [51] and alleviates SARS-CoV-2-triggered cytokine storm, sepsis, thrombosis, and lung fibrosis [52] (Figure 3). In addition, EGCG decreases the severity of Omicron-related COVID-19 symptoms in both elderly patients and patients with metabolic syndrome by downregulating GRP78 expression or promoting hyperinsulinemia remission [43].

Despite its broad-spectrum antiviral activity, favorable safety profile, and multi-organ protective effects, EGCG demonstrated poor oral bioavailability (*F*) in both rats (*F* = 0.1%) and humans (*F* = 0.3%) [53]. Accordingly, the development of an EGCG throat spray as a potential therapeutic strategy targeting Omicron infection should be further explored in the clinical setting.

## 3. Myricetin—A Waxberry-Derived Covalent M^pro^ Inhibitor Suitable for Lead Optimization

Myricetin is a well-known nutritional supplement that can be isolated from “medicine food homology” plants, such as *Myrica rubra*, *Ampelopsis grossedentata*, *Malus domestica*, and *Cistus monspeliensis* [54]. Specifically, vine tea (*A. grossedentata*), which has myricetin as the main bioactive ingredient, received approval as a functional food ingredient in 2013 and is traditionally consumed worldwide owing to its health-promoting effects and pleasurable taste [55]. Myricetin, a natural dietary flavonol, has numerous pharmacological effects, including improving bleomycin-induced pulmonary fibrosis via the targeting of HSP90β [56], combating methicillin-resistant *Staphylococcus aureus*-related lethal pneumonia by inhibiting caseinolytic peptidase P [57], ameliorating brain injury and neurological deficits via nuclear factor erythroid 2-related factor 2 activation [58], enhancing immunomodulatory functions [59], and mitigating hepatic fibrosis via the inhibition of the TREM-1-TLR2/4-MyD88 signaling pathway [60]. Myricetin is also an antiviral drug with low toxicity that can treat a wide variety of viral infections in vitro, including Ebola virus (IC_50_ = 2.7 μM) [61], Marburg virus (IC_50_ = 25.5 μM) [62], infectious bronchitis virus (IC_50_ = 10.6 μM) [63], HIV-1 virus (IC_50_ = 7.6 μM) [64], African swine fever virus (IC_50_ = 8.4 μM) [65], Bourbon virus (IC_50_ = 2.2 μM) [66], and herpes simplex virus (IC_50_ = 1.6 μM) infections [67].

Myricetin is an ideal candidate for research targeting SARS-CoV-2 infection. SARS-CoV-2 helicase (NSP13), a highly conserved non-structural protein possessing RNA helicase and 5′-triphosphatase activities, is a promising target for the development of novel anti-SARS-CoV-2 drugs [68]. Corona et al. [69] showed that myricetin inhibits NSP13 helicase-associated activity, with an IC_50_ value of 0.41 µM. Moreover, Xiao et al. [70] reported that myricetin effectively inhibits SARS-CoV-2 replication in vitro by targeting M^pro^ (IC_50_ = 3.68 μM; no cytotoxicity was detected with concentrations of up to 50 μM). Further analysis revealed that the 3′- and 4′-hydroxyl groups of myricetin form hydrogen bonds with Phe140 and Glu166 of M^pro^, while the chromone ring of myricetin forms π–π stacking interactions with His41, which stabilizes its binding in the catalytic center of M^pro^. COVID-19 is primarily an inflammatory disease [71]. In a different study, the same authors [70] revealed that myricetin can effectively inhibit lung inflammation by suppressing inflammatory cell infiltration and the secretion of inflammatory factors (IL-6, IL-1α, TNF-α, and IFN-γ). Similarly, Kato et al. [37] reported that myricetin inhibits the synthesis of SARS-CoV-2 M^pro^, with an IC_50_ value of 0.90 μM, while Kuzikov et al. [72] revealed that myricetin displays excellent anti-SARS-CoV-2 potency in vitro (IC_50_ = 0.22 μM). The authors [72] further reported the X-ray crystal structure of M^pro^ complexed with myricetin at a resolution of 1.77 Å (PDB ID: 7B3E), which unambiguously revealed that the mechanism of action involves the formation of a covalent bond between Cys145 and the 2′ position of myricetin.

**Figure 4:**
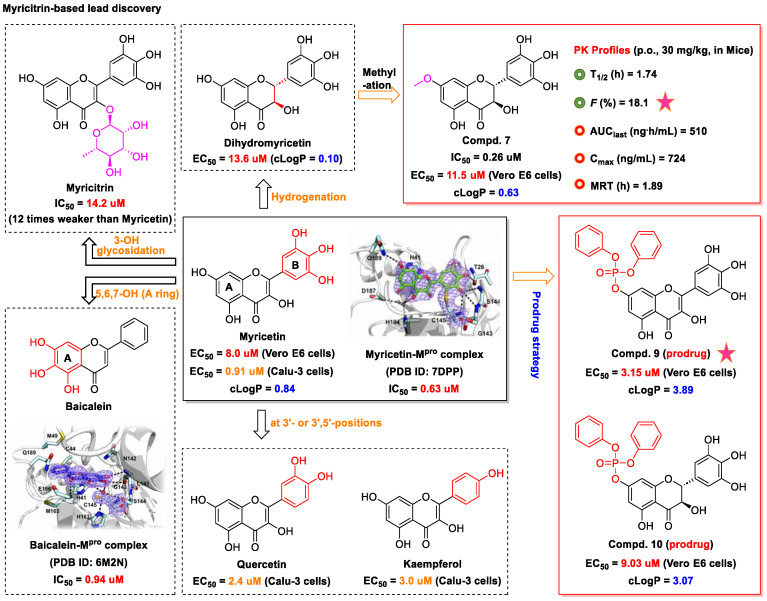
Myricetin-based lead discovery and optimization. Myricetin is a waxberry-derived covalent M^pro^ inhibitor suitable for lead optimization.

Although myricetin has broad-spectrum antiviral potential without serious adverse effects, its use is limited due to its poor solubility and low oral bioavailability (<10%) [73,74]. Consequently, the development of myricetin prodrugs or oral derivatives, with enhanced bioavailability and membrane permeability, has been proposed as an alternative tactic for drug design (Figure 4). The pyrogallol group of the myricetin B ring, acting as an electrophile, is covalently bound to Cys145, helping to maintain a strong anti-SARS-CoV-2 potential [18]. Chaves et al. [75] evaluated a series of structurally similar natural flavonoids, including myricetin, and found that the presence of fewer hydroxyl groups in ring B of these flavonols (myricetin, three hydroxyl groups; quercetin, two hydroxyl groups; and kaempferol, one hydroxyl group) was correlated with reduced anti-SARS-CoV-2 activity in Calu-3 cells, with EC_50_ values of approximately 0.91, 2.40, and 3.02 μM, respectively. Notably, when the pyrogallol group of the myricetin B ring was transferred to the A ring, the binding mode of myricetin to M^pro^ was fundamentally changed.

Myricetin and another M^pro^ inhibitor, baicalein, possess pyrogallol groups, but their modes of action and their structural determinants of protease binding are different [18]. An examination of the crystal structure of the myricetin-M^pro^ complex (2.1 Å, PDB ID: 7DPP) revealed the presence of a covalent bond between the sulfur atom of Cys145 of M^pro^ and the C6′ atom of the pyrogallol group of myricetin (Figure 4) [18]. Several other interactions were identified, such as hydrogen bonding between the hydroxyl groups of myricetin and Gly143, Ser144, Cys145, and Thr26; π–π stacking interactions between the chromone moiety of myricetin and His41; and the formation of hydrogen bonds between the chromone moiety and Glu189, His164, His41, and Asp187 of M^pro^. In contrast, observation of the crystal structure of baicalein complexed with M^pro^ (2.2 Å, PDB ID: 6M2N) revealed that baicalein forms multiple interactions (π–π stacking, hydrogen bonds, and hydrophobic interactions) with specific residues of M^pro^, rather than covalently blocking the catalytic Cys145 residue (Figure 4) [18].

**Figure 5:**
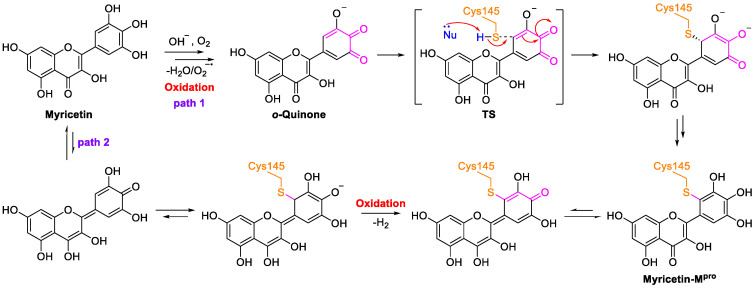
Possible mechanism of myricetin oxidation.

In terms of structure, the pyrogallol group of the B rings of flavanols can be easily oxidized to form orthoquinone, which covalently binds to Cys145 of M^pro^ (Figure 5) [73]. These studies present a starting point for structure-based lead identification and optimization of flavanol-based compounds.

**Figure 6:**
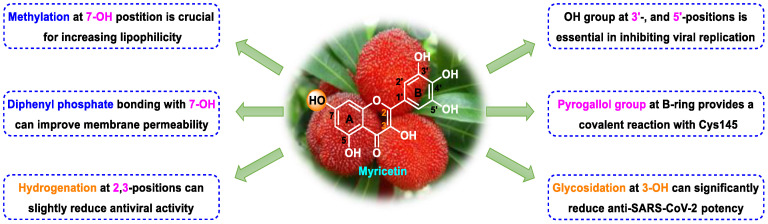
Structure–activity relationships of myricetin derivatives as leads for SARS-CoV-2 treatment.

Furthermore, Xiong et al. [76] found that myricetin (IC_50_ = 1.2 μM) and its glycoside myricitrin (IC_50_ = 14.2 μM) can inhibit SARS-CoV-2 replication by covalently binding to the biothiols of M^pro^ in a dose- and time-dependent manner. Nevertheless, the antiviral activity of myricitrin (IC_50_ = 14.2 μM) is 11.8-fold weaker than that of myricetin (IC_50_ = 1.2 μM), which demonstrates the pivotal role of the free C3 hydroxyl group in promoting the binding of myricetin to M^pro^ through hydrogen bonding. Besides that, dihydromyricetin, a *trans*-conformation of myricetin (hydrogenation of its C2=C3 double bond), displays weaker inhibitory activity compared to that of myricetin (IC_50_: 1.14 vs. 0.63 μM; EC_50_: 13.56 vs. 8.00 μM) [18]. This is likely because the presence of the C2=C3 bond increases electron delocalization in the A–C ring of myricetin and enhances the stability of π-conjugation with His41. Together, these results demonstrate that the 3-OH and 3′,4′,5′-OH moieties of myricetin are essential for its potent anti-SARS-CoV-2 activity (Figure 6) [18].

Lipophilicity (cLogP) is an important physicochemical parameter influencing oral absorption and pharmacokinetic properties [77,78]. Su et al. [18] found that the addition of an alkyl group (methyl, ethyl, isoamyl, or cyclopentylmethyl) to the 7-OH of myricetin can increase the lipophilicity of the resulting compound relative to that of myricetin, thereby enhancing the inhibition of the enzymatic activity of SARS-CoV-2 M^pro^. An analysis of the structure–activity relationship of the derived compounds suggested that the smaller methyl group may bind more strongly to a specific but small sub-pocket within M^pro^ compared to the other, larger alkyl groups. Subsequently, a methyl group was introduced to the 7-OH of dihydromyricetin, yielding compound **7**, which could significantly inhibit viral replication (IC_50,_ 0.26 μM; EC_50_, 11.5 μM) (Figure 4) [18]. When administered orally to mice (30 mg/kg compound **7** per day), this compound showed an improved pharmacokinetic profile compared to that of myricetin (*T*_max_, 1.74 vs. 0.50 h; *C*_max_, 724 vs. 8.59 ng/mL; AUC_last_, 510 vs. 6.07 ng·h/mL; MRT, 1.89 vs. 0.84 h; and *T*_1/2_, 1.74 vs. 0.44 h for compound **7** and myricetin, respectively). Compound **7** displayed acceptable oral bioavailability (*F* = 18.1%), given that compounds with an oral bioavailability greater than 10% have potential for development as oral drugs [18]. The current data support the further optimization of 7-*O*-methylmyricetin-based oral inhibitors for COVID-19 treatment. Prodrugs have better pharmacokinetic properties, and their hidden pharmacological activities can be recovered after biotransformation in vivo, thereby representing an excellent option for the design of COVID-19-targeting drugs [79,80]. To improve the aqueous solubility and the membrane permeability of myricetin, compound **9** was produced via *the* introduction of diphenyl phosphate at the 7-OH moiety of myricetin (Figure 4) [18]. Compared to myricetin, compound **9** displayed significantly increased lipophilicity (cLogP, 3.89 vs. 0.84) and better inhibitory activity against SARS-CoV-2 replication (EC_50_, 3.15 vs. 8.00 μM). Similarly, the same diphenyl phosphate group was added to the 7-OH of dihydromyricetin, yielding compound **10**, with an EC_50_ against SARS-CoV-2 replication superior to that of dihydromyricetin (EC_50_, 9.03 vs. 13.6 μM) [18]. Myricetin prodrugs are still experimental, and further studies are needed to demonstrate their safety and efficacy.

## 4. Other Anti-SARS-CoV-2 Natural Dietary Flavonoids in Development for Treating SARS-CoV-2 Infection

Natural dietary flavonoids can make a substantial contribution to mitigating the effects of the COVID-19 pandemic given their good safety profile and antiviral activities. In addition to the abovementioned bioactive compounds, other natural dietary flavonoids, shown in Table 1, have demonstrated strong anti-SARS-CoV-2 activity in vitro, and thus can serve as a starting point for further drug development for the treatment of COVID-19.

## 5. Conclusions and Future Directions

The COVID-19 pandemic represented an unprecedented global health crisis. Functional foods and nutritional supplements are excellent complements to vaccines and therapeutics. They encompass a large and rich library of natural bioactive products, some of which are likely to exhibit anti-COVID-19 therapeutic potential. Natural dietary flavonoids are conceptually attractive as treatment options in response to outbreaks; however, their implementation is challenging. To obtain maximal benefits, several aspects should be considered to facilitate the development of natural dietary-bioactive-product-based drugs.

First, additional research directly related to SARS-CoV-2 and its variants is urgently needed to clarify the effectiveness of the above-mentioned flavonoids. Natural dietary bioactive flavonoids must be rigorously evaluated in in vitro, animal model, and clinical studies rather than relying only on virtual screening, network pharmacological prediction, or machine learning models, especially when their targets or mechanisms of action are unknown. For instance, designing controlled clinical trials may help elucidate any additional effects of these compounds. Natural dietary flavonoids may make a marked contribution toward controlling hyperinflammatory responses and preventing lung injury. Nevertheless, the underlying mechanisms require further exploration and systematic clarification.

Secondly, combination therapy could contribute to addressing potential drug resistance associated with emerging variants. Evolution and the associated increase in selection pressures may yield SARS-CoV-2 variants that are resistant to antiviral therapies. While resistant viruses could rapidly emerge in the presence of a single agent, the appearance of escape mutations against a combination of compounds, displaying different mechanisms of action and resistance profiles, is less likely. Multi-target therapeutic modalities (mixtures of natural dietary flavonoids, similar to drug cocktails) with broad variant activity could further improve the effectiveness against globally circulating SARS-CoV-2 variants and mitigate the emergence of new escape mutants. Importantly, however, monitoring for potential drug–drug interactions is essential in the development of combination therapies. In parallel, lead optimization of multi-target dietary compounds (e.g., EGCG targeting SARS-CoV-2 NSP15, S protein, and M^pro^) could potentially provide candidates for COVID-19 treatment.

Thirdly, alternative routes of administration (e.g., oral or inhalation) have the potential to maximize clinical benefit. Compared to the intravenous route, oral or inhalation administration can help address compliance issues given that the associated medications are less likely to require refrigeration, while also avoiding the use of needles. Oral administration can maximize clinical benefits by shortening the duration of COVID-19 and reducing acute post-sequelae symptoms of SARS-CoV-2 infection. Inhalation administration facilitates the direct delivery of antiviral agents to the primary site of infection, reducing systemic exposure to drugs and their metabolites, thereby minimizing systemic side effects.

In summary, to address current limitations associated with the use of dietary components for the treatment of SARS-CoV-2 infection, a mixture of factors, including combination therapy, lead optimization, and drug delivery, must be considered.

## Figures and Tables

**Table 1:** Other natural dietary flavonoids for treating SARS-CoV-2 infection in vitro.

No.	Name	Species	Structure	EC_50_ or IC_50_ (μM)	Target or Mechanism	Refs.
1	Hesperidin	*Citrus sinensis*	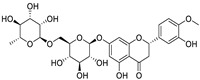	13.25	ACE2, M, S, and RBD proteins	[81,82]
2	Ugonin J	*Helminthostachys zeylanica*	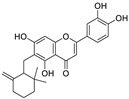	2.38	M^pro^	[83]
3	Epicatechin-3-*O*-gallate	*Camellia sinensis* var. *sinensis*	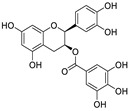	5.21	M^pro^	[84,85]
4	Catechin-3-*O*-gallate	*Senegalia catechu*	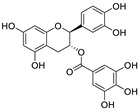	2.98	M^pro^	[84]
5	Procyanidin B_2_	*Punica granatum*	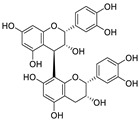	75.3	M^pro^	[84,86]
6	Osajin	*Maclura pomifera*	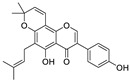	3.87	N protein, nsp16, and nsp13	[87,88]
7	(+)-Gallocatechin	*Musa Cavendish*	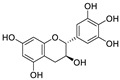	13.14	M^pro^	[89,90]
8	Apigenin-7-*O*-glucoside	*Achillea millefolium* L.	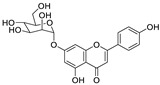	0.074	M^pro^	[91,92]
9	Naringenin	*Citrus reticulata*	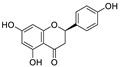	0.092	M^pro^, NSP12, NSP7, NSP8, and NSP3	[91,93]
10	etc-pyrrolidinone C and D	*Camellia sinensis*	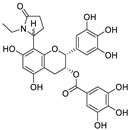	0.90	M^pro^	[94]
11	(−)-epicatechin 3-O-caffeoate	*Camellia sinensis*	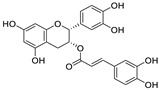	1.58	M^pro^	[94]
12	Quercetin	*Citrus reticulata* Blanco	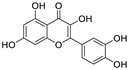	18.2	M^pro^	[95,96]
13	3,8′-biapigenin	*Forsythia suspensa*	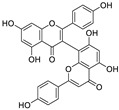	13.0	M^pro^, protein disulfide isomerase	[97]
14	PGHG	*Penthorum chinense* Pursh	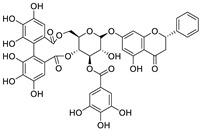	6.5	M^pro^, protein disulfide isomerase	[97]
15	Luteolin	*Taraxacum antungense* Kitag	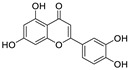	11.81	M^pro^, RBD-ACE2	[98,99,100]
16	Isorhamnetin	Sea buckthorn	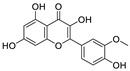	8.42/2.51	M^pro^	[98,101]
17	Baicalein	*Scutellaria baicalensis* Georgi	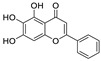	0.39	M^pro^, RdRp	[102,103]
18	Scutellarein	*Scutellaria baicalensis* Georgi	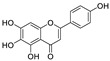	5.8	M^pro^	[102]
19	Proanthocyanidin	Grape seed	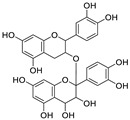	25.90/21.02	M^pro^, and RdRp	[104,105]
20	Theaflavin 3-gallate	Black tea	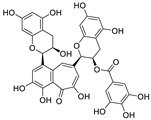	18.48	M^pro^, S protein	[106,107]
21	Theaflavin	Black tea	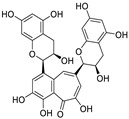	22.22	M^pro^	[106]
22	3′,5′,5,7-tetrahydroxy-6-methoxyflavanone	*Helichrysum bracteatum*	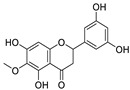	5.565	M^pro^	[108]
23	Kaempferol	*Canavalia ensiformis* L.	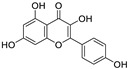	34.46	M^pro^, PL^pro^	[109,110]
24	Amentoflavone	*Nandina domestica*	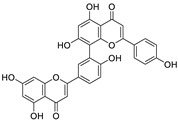	13.0	PL^pro^, RBD-ACE2	[111,112]
25	Scutellarein	*Scutellaria baicalensis*	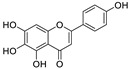	5.80	M^pro^	[102,113]
26	Epicatechin gallate	*Fagopyrum esculentum*	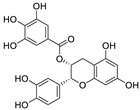	12.5	M^pro^	[36,114,115]
27	Schaftoside	*Prosopis alba* cotyledons	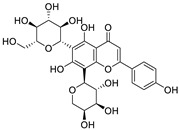	11.83	M^pro^ and PL^pro^	[116]
28	Astilbin	*Smilax glabra* Roxb.	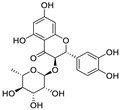	7.92	M^pro^	[117]
29	Astragalin	*Nelumbo nucifera*	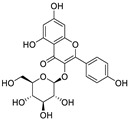	0.13	M^pro^	[117,118]
30	Apigenin	*Apium Graveolens* L.	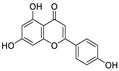	5.21	M^pro^	[119,120]
31	Baicalin	*Scutellaria baicalensis*	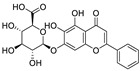	8.8	RdRp and M^pro^	[121,122]
32	Rhodiosin	*Rhodiola rosea*	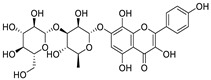	0.48	NSP13	[123]

## Data Availability

No data were used for the research described in the article.
